# High Dose of Intravenous Allogeneic Umbilical Cord-Derived Mesenchymal Stem Cells (CLV-100) Infusion Displays Better Immunomodulatory Effect among Healthy Volunteers: A Phase 1 Clinical Study

**DOI:** 10.1155/2020/8877003

**Published:** 2020-09-28

**Authors:** Sze-Piaw Chin, Mohd-Yusoff Mohd-Shahrizal, Mohd-Zuhar Liyana, Kong Yong Then, Soon Keng Cheong

**Affiliations:** ^1^NSCMH Medical Centre, Seremban, Negeri Sembilan, Malaysia; ^2^Cytopeutics, Cyberjaya, Malaysia; ^3^Universiti Tunku Abdul Rahman, Bandar Sungai Long, Selangor, Malaysia

## Abstract

**Background:**

Mesenchymal stem cells (MSCs) express growth factors and other cytokines that stimulate repair and control the immune response. MSCs are also immunoprivileged with low risk of rejection. Umbilical cord-derived MSCs (UCMSCs) are particularly attractive as an off-the-shelf allogeneic treatment in emergency medical conditions. We aim to determine the safety and efficacy of intravenous allogeneic infusion of UCMSCs (CLV-100) by Cytopeutics® (Selangor, Malaysia) in healthy volunteers, and to determine the effective dose at which an immunomodulatory effect is observed. *Methodology*. Umbilical cord samples were collected after delivery of full-term, healthy babies with written consent from both parents. All 3 generations (newborn, parents, and grandparents) were screened for genetic mutations, infections, cancers, and other inherited diseases. Samples were transferred to a certified Good Manufacturing Practice laboratory for processing. Subjects were infused with either low dose (LD, 65 million cells) or high dose (HD, 130 million cells) of CLV-100 and followed up for 6 months. We measured cytokines using ELISA including anti-inflammatory cytokines interleukin 1 receptor antagonist (IL-1RA), interleukin 10 (IL-10), pro-/anti-inflammatory cytokine interleukin 6 (IL-6), and the proinflammatory cytokine tumor necrosis factor-alpha (TNF-*α*).

**Results:**

11 healthy subjects (LD, *n* = 5; HD, *n* = 6; mean age of 55 ± 13 years) were recruited. All subjects tolerated the CLV-100 infusion well with no adverse reaction throughout the study especially in vital parameters and routine blood tests. At 6 months, the HD group had significantly higher levels of anti-inflammatory markers IL1-RA (705 ± 160 vs. 306 ± 36 pg/mL; *p* = 0.02) and IL-10 (321 ± 27 vs. 251 ± 28 pg/mL; *p* = 0.02); and lower levels of proinflammatory marker TNF-*α* (74 ± 23 vs. 115 ± 15 pg/mL; *p* = 0.04) compared to LD group.

**Conclusion:**

Allogeneic UCMSCs CLV-100 infusion is safe and well-tolerated in low and high doses. Anti-inflammatory effect is observed with a high-dose infusion.

## 1. Introduction

Mesenchymal stem cells (MSCs) are multipotent fibroblast-like cells that reside in various tissues of the human body. MSCs have the capacity to regenerate and replicate as well as to differentiate into various specialized cells and tissues in the body, including chondrocytes, adipocytes, osteocytes, and neuron-like cells [1–3]. The self-renewal and multilineage potentials of MSCs in providing new cells for tissue repair by replacing the damaged cells suggest its therapeutic potentials in tissue regeneration [2, 4–8].

Several studies have reported that the mechanism of MSCs in repairing tissue damage is associated to their immunomodulatory properties rather than its capacity for differentiation [9, 10]. One of MSCs' vital biological function, the immunomodulation, provides MSCs with the ability to migrate and adhere to any injury or inflammation sites found in the body and thereby interact with various immune cells such as T cells, B cells, natural killer cells, dendritic cells, neutrophils, and macrophages before evoking effective immune responses to ameliorate the intense inflammatory reaction of the injured site via direct cell-cell contact mechanism and/or the release of soluble inducible factors [11–13].

MSCs can be isolated from various tissues including bone marrow, peripheral blood, adipose tissue, cord blood, and umbilical cord. Recent studies have shown that MSCs derived from human umbilical cord (UCMSCs) possess several advantages compared to MSCs isolated from other tissues, including high-proliferation and self-renewal capacity and multilineage differentiation capability. Umbilical cord is considered as a medical waste, and the collection of UC-MSCs is noninvasive which eliminates any ethical concern from its collection. [14, 15]. In addition, UCMSCs possess low immunogenicity allowing them to be utilized in allogeneic transplantation without any rejection and thereby providing a new approach for the treatment of autoimmune diseases [16].

Consequently, UCMSCs have been developed as an “off-the-shelf” cell therapy for a variety of diseases especially in autoimmune diseases. Clinical studies in graft-versus-host disease (aGVHD) have demonstrated that UCMSCs dramatically improved the patients' conditions with no adverse effects and no evidence of cancer recurrence throughout the trial period ([17, 18]). Moreover, UCMSCs treatment in active systemic lupus erythematosus (SLE) resulted in amelioration of the disease activity, serologic changes, and stabilization of proinflammatory cytokines in the patients [19].

The production of UCMSCs cells products from manufacturing methods must be tested for its safety prior to be used as therapeutic agents in cell therapy ([20]). Therefore, this Phase 1 clinical study was conducted to establish a new UCMSCs cell line (CLV-100) by assessing the safety and efficacy of intravenous allogeneic infusion of our manufactured UCMSCs (CLV-100) among healthy volunteers. This study also sought to compare the immunomodulatory effect of different dosage of CLV-100 between high-dose and low-dose infusion in healthy volunteers based on several clinical assessments and measurements of changes in systemic biomarkers. The findings of this study will act as a guideline and benchmark for future CLV-100 clinical research.

## 2. Materials and Methods

### 2.1. Study Design

This is an open-label nonrandomized Phase 1 study assessing the safety and efficacy of CLV-100 infusion among 11 healthy subjects recruited at NSCMH Medical Centre in Seremban, Malaysia. The subjects were divided into 2 groups; low-dose group received 65 million cells (equivalent to about 1 million cells per kg body weight) (LD, *n* = 5), while high dose group received 130 million cells (equivalent to about 2 million cells per kg body weight) (HD, *n* = 6) of allogeneic infusion of CLV-100. This study was approved by the Medical Research and Ethics Committee (MREC) Ministry of Health Malaysia (NMRR-13-1152-17400) and monitored by independent Data Safety and Monitoring Board (DSMB). All subjects provided written informed consent before participating in the study. The inclusion and exclusion criteria were listed in Table 1.

### 2.2. Establishing UCMSCs Culture

Umbilical cord samples were collected after delivery of full-term, healthy babies with written consent from both parents. All 3 generations (newborn, parents, and grandparents) were screened for genetic mutations, infections, cancers, and other inherited diseases before the samples were transferred to the laboratory for processing. All cell processing was done in a certified Good Manufacturing Practice (GMP) laboratory in accordance with Malaysia Guidelines for Stem Cell Research and Therapy as published previously [4, 5]. Isolation and culturing have been established and reported previously [21]. The high-quality umbilical cord was digested, and MSCs were isolated based on adherence to flask's surface. The cells then were expanded in proprietary growth medium kept in 37°C, 5% CO_2_, and 95% air incubator. After three days, nonadherent cells were discarded and replaced with new growth medium until it reached confluence. Then, the MSCs were cultured in new flasks until the required cell number was achieved. The first few early passages of the cells were cryopreserved and served as a seed for future use. For this study, cells were thawed and expanded from the seed up until Passage 6. Throughout the process, UCMSCs were tested for quality control purposes including immunophenotyping, differentiation assays, as well as to confirm the absence of bacterial, fungal, and mycoplasma contamination.

### 2.3. Infusion of UCMSCs, Monitoring, and Follow-Up

On the day of CLV-100 infusion, the eligible subjects registered to a medical centre as outpatients. The subjects underwent a routine physical examination, and their vital signs were measured to ensure they were fit and suitable for CLV-100 infusion. Once the subjects were confirmed fit for CLV-100 infusion, a cannula was placed in the subjects' vein. Before CLV-100 infusion, 200 mL of normal saline was infused into subjects for 0.5-1 hours. While waiting for normal saline infusion, 65 million CLV-100 for LD group and 130 million cells for HD group were thawed, washed, and resuspended in 200 mL of normal saline before being infused intravenously to the subjects for 1 hour. Upon completion, 50 mL of normal saline solution was infused to keep the vein open. All standard precautions for intravenous procedure were observed according to routine and standard practice at the medical centre. The subjects were monitored for vital sign and adverse event (AE) (if any) every 15 minutes during infusion and later on an hourly basis for a minimum of 6 hours in the medical centre. We followed the Good Clinical Practice (GCP) guidelines of the International Council for Harmonization (ICH) in defining our AE. The classifications of AE in this study include any untoward medical occurrence in the study subject administered with CLV-100 which may or may not related to the Investigational Product (IP). The monitored AE included but not limited to fever, headache, injection site swelling, or pain. The subjects were discharged if there were no other complications observed after the monitoring period.

Subjects were required to do the 4 times of follow-up (2, 30, 90, and 180 days) postinfusion. During all follow-up, 20 mL blood was withdrawn from the subjects for blood analysis. Subjects were required to immediately inform the medical centre if there is any AE or severe adverse event (SAE).

### 2.4. Outcome Measures

Baseline data were collected from each subject prior to CLV-100 infusion and information regarding subject particulars; demographic data and medical history were properly recorded. Several clinical assessments were performed at baseline (before CLV-100 infusion) and during postinfusion follow-ups at 2, 30, 90, and 180 days, including routine blood tests, hypersensitivity tests (specifically white cell count, subfraction, and immunoglobulin E), vital signs, lung function tests specifically in the ratio of forced expiratory volume in 1 second to forced vital capacity (FEV1/FVC) via spirometry, renal function tests, liver function tests, full blood count, level of the proinflammatory and the anti-inflammatory markers such as high-sensitivity C-reactive protein (hs-CRP), and albumin globulin ratio (A/G), respectively, as well as cytokines, to examine any changes in the results in each of the subjects.

The primary endpoint of this study was to evaluate the presence or absence of allergic reaction to the infusion, sepsis, organ failure (clinically apparent or subclinical), hospitalization, cancers, and death, as well as any changes in clinical, functional parameters and blood tests during the 6 months follow-up period.

### 2.5. Detection of Cytokines and Growth Factors with ELISA

Serum of every subject during day 0, day 2, day 7, day 30, and day 180 postinfusion was collected and kept frozen at -80°C to allow batch analysis at the end of the study. The anti-inflammatory cytokines including interleukin-10 (IL-10), interleukin-1-receptor antagonist (IL-1RA), and prostaglandin E2 (PGE2); proinflammatory cytokines such as interleukin-6 (IL-6) and tumor necrosis factor-alpha (TNF-*α*); as well as growth factors including transforming growth factor-beta (TGF-*β*), vascular endothelial growth factor (VEGF), and hepatocyte growth factor (HGF) were detected and measured with enzyme-linked immunosorbent assay (ELISA) kits (R&D System, USA) in duplicates according to manufacturer's instructions.

### 2.6. Statistical Analysis

Data analysis was performed by IBM SPSS Statistic v23.0 software (SPSS, Inc., Armonk, NY, USA). Missing data on the primary efficacy variable had their data imputed by the method of Last Observation Carried Forward (LOCF), and the data were presented as means ± SD. The safety of intravenous infusion of CLV-100 towards the subjects was assessed via descriptive statistic analysis. Differences in side effects and blood test between the group of low dose and high dose were calculated using Fisher's exact test (for categorical data) and Mann–Whitney test (for numerical data). As for the efficacy analysis, Mc Nemar test (for categorical data) and Wilcoxon signed rank test (for numerical data) were used to assess the difference (if any) between pre- and post-CLV-100 infusion. It was considered statistically significant when the value of *p* < 0.05.

## 3. Results

### 3.1. Baseline Assessments

Throughout the study period, 11 healthy volunteers (male: 4, female: 7) were screened and recruited between May 2017 and January 2018 into the study. As shown in Table 2, the mean age of the subjects during baseline was 55 ± 13 years old. About 1.1 ± 0.2 million cells per kg and 2.1 ± 0.3 million cells per kg were infused into LD subjects and HD subjects, respectively. There were no significant differences were observed in all clinical routine parameters between the two groups except HD subjects have higher but normal white blood cells (WBC) count (7.3 ± 0.9 vs. 6.0 ± 0.5 × 10^9^/L; *p* = 0.03) and alpha-fetoprotein (AFP) level (4.2 ± 2.3 vs. 1.7 ± 0.4 ng/mL; *p* = 0.04) compared to LD subjects. We have further looked into individual WBC and AFP parameters. All subjects (either LD or HD) were within the normal range (4.0 − 11.0 × 10^9^/L for WBC and less than 10 ng/mL for AFP). Further on that, from 30 to 180 days (to the end of the study) postinfusion follow-up, no statistical significant differences were observed in WBC and AFP parameters.

### 3.2. Tolerability, Hypersensitivity and Adverse Reactions

Similar clinical assessments were examined throughout the 6-months follow-up among all recruited subjects in both groups to assess the safety of allogeneic CLV-100 infusion as shown in Table 3 and Table 4. All subjects tolerated the CLV-100 infusion well. There were no significant different changes in vital signs variables before, during, and after the CLV-100 in both groups. There was a small but significant increase in hemoglobin (13.8 ± 1.2 vs. 14.4 ± 1.3 g/dL; *p* = 0.04) and MCV (86.0 ± 2.8 vs. 88.2 ± 3.9 fl; *p* = 0.02) at 6 months in HD group.

Specifically, the immunoglobulin E (IgE), which is normally raised in hypersensitivity reactions, remained low within the normal range for both LD (23.3 ± 19.2 vs. 22.1 ± 12.0 IU/mL; *p* = 0.14) and HD (49.7 ± 54.5 vs. 50.2 ± 53.3; *p* = 0.79) groups throughout the study although the values are not significant. Similarly, there was no increase or decrease in total white cell count or its subfractions after infusion. For the lung function test, no significant difference was observed in both FEV1 (2.4 ± 0.4 vs. 2.3 ± 0.2 L; *p* = 0.79) and FEV1/FVC (86 ± 7 vs. 83 ± 6%; *p* = 0.95) levels throughout the follow-up period in HD group. In addition, there were no reported AE or SAE among all subjects throughout the 6 months follow-up period.

### 3.3. Immunomodulatory Effect of CLV-100 by Measurement of Cytokines

The immunomodulatory effect of CLV-100 infusion in the healthy volunteers of both dosage groups was measured based on the changes in systemic biomarkers detected in the subjects' collected sera. The cytokines levels of the subjects in LD and HD groups were compared between baselines and postinfusion.

**Figure 1:**
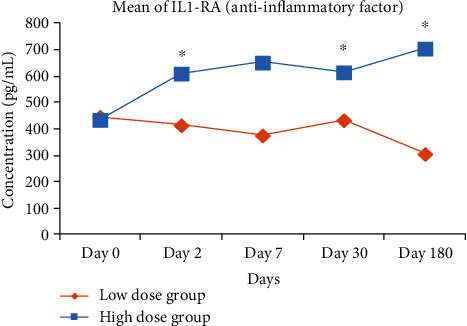
Serum level of IL1-RA measured in every follow-up visits for all subjects in the LD and HD group. The anti-inflammatory IL-1RA was significantly elevated in HD subjects from day 2 to day 180 relative to baseline. Statistical significance of biomarkers between each follow-up visits was assessed by using the Wilcoxon signed-rank test. ^∗^Significant value at *p* < 0.05 when compared with baseline.

In the HD group, the serum level of anti-inflammatory IL-1RA was significantly elevated from day 0 to day 2 (436 ± 128 vs. 610 ± 176 *pg*/*mL*; *p* = 0.03), day 30 (436 ± 128 vs. 615 ± 148 *pg*/*mL*; *p* = 0.03), and day 180 (436 ± 128 vs. 705 ± 160 *pg*/*mL*; *p* = 0.03) postinfusion as depicted in Figure 1. The remaining cytokines (IL-10, IL-6, PGE2, and TNF-*α*) did not show any statistical significant mean changes from baseline throughout the monitoring period. In the LD group, the serum levels of IL-10, IL-1RA, IL-6, PGE2, and TNF-*α* did not show any significant changes within group for each of the follow-up visits as compared to baseline.

**Figure 2:**
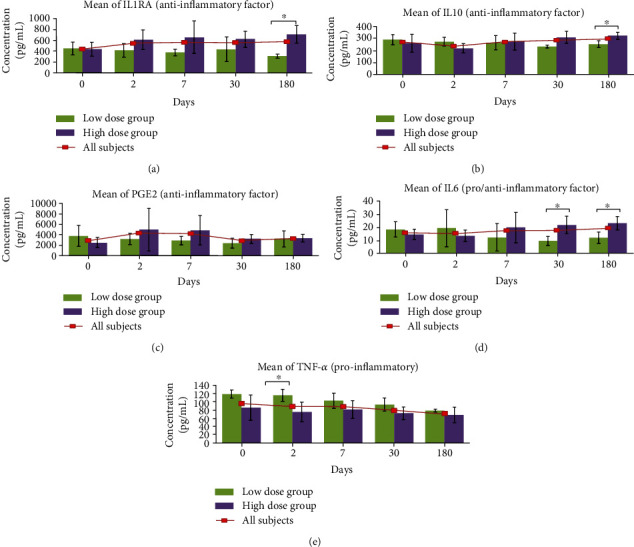
Evaluation of serum levels of cytokines, IL-1RA, IL-10, PGE2, IL-6, and TNF-*α*, for subjects between LD and HD group. Only serum levels of cytokines IL-1RA, IL-10, IL-6, and TNF-*α* were found to have significant changes between both groups. Statistical significance of biomarkers between LD and HD groups were assessed by using the Mann–Whitney test. ^∗^Significant value at *p* < 0.05.

The serum cytokines levels at different time points were also compared between LD and HD group (Figure 2). The serum levels of anti-inflammatory factors IL1-RA (705 ± 160 vs. 306 ± 36 pg/mL; *p* = 0.02) and IL-10 (321 ± 27 vs. 251 ± 28 pg/mL; *p* = 0.02) were significantly greater at day 180 postinfusion in HD group than in LD group. In addition, the serum level of proinflammatory factor TNF-*α* was significantly lower at day 2 (74 ± 23 vs. 115 ± 15 pg/mL; *p* = 0.04) after infusion in the HD group compared to the LD group. Meanwhile, the serum level of IL-6, which has both pro- and anti-inflammatory properties, was significantly higher in HD group at day 30 (22 ± 7 vs. 10 ± 4 pg/mL; *p* = 0.05) postinfusion in relative to LD group, and similar trend was observed as the follow-up continues at day 180 (23 ± 5 vs. 12 ± 4 pg/mL; *p* = 0.02). Finally, there was no significant difference of PGE2 level observed between both groups.

### 3.4. Albumin Globulin Ratio as a Marker of Anti-Inflammatory State

**Figure 3:**
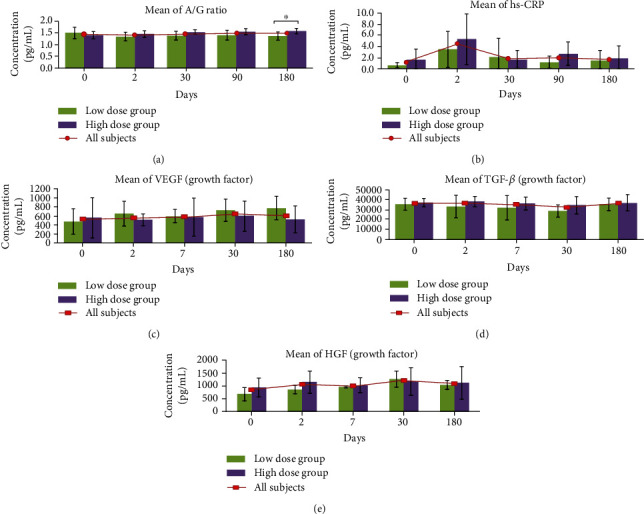
Evaluation of serum levels of A/G ratio, hs-CRP as well as VEGF, TGF-*β*, and HGF for subjects between LD and HD group. Only serum levels of A/G were found to have significant changes between both groups. Statistical significance of the biomarkers between LD and HD groups were assessed by using the Mann–Whitney test. ^∗^Significant value at *p* < 0.05.

In the HD group, albumin/globulin (A/G) ratio (1.4 ± 0.2 vs. 1.6 ± 0.1; *p* = 0.01) was significantly elevated, with a corresponding significant drop of globulin (31.3 ± 1.6 vs. 27.8 ± 1.6 g/L; *p* = 0.01) level was observed over 6 months period. When comparing between both groups, the HD subjects have higher A/G ratio compared to the LD subjects at 6 months (1.6 ± 0.1 vs. 1.4 ± 0.2; *p* = 0.04) post-CLV-100 infusion as shown in Figure 3(a).

### 3.5. Hs-CRP as a Marker of Inflammation, Repair, and Healing

In LD group, high-sensitivity C-reactive protein (hs-CRP) reading was significantly elevated at 2 days postinfusion (0.6 ± 0.5 vs. 3.5 ± 3.2 mg/L; *p* = 0.04) but dropped continuously over 6 months follow-up (1.5 ± 1.8 mg/L; *p* = 0.09). A similar trend was detected among HD subjects where the hs-CRP value significantly raised at 2 days postinfusion (1.6 ± 1.9 vs. 5.3 ± 4.5 mg/L; *p* = 0.03) and subdued tremendously at 6 months (1.8 ± 2.3 mg/L; *p* = 0.04). The hs-CRP serum level in both groups was depicted in Figure 3(b).

### 3.6. Change in Serum Growth Factors

When the growth factors level of the subjects in LD and HD groups collected sera were measured via systemic biomarkers, all growth factors, which are VEGF (Figure 3(c)), TGF-*β* (Figure 3(d)), and HGF (Figure 3(e)), did not show any statistical significant mean changes from baseline throughout the monitoring period as well between both groups.

## 4. Discussion

The main objective of this clinical study was to determine the safety of allogeneic intravenous CLV-100 infusion among healthy volunteers with different doses. Based on the results, there was no complication that occurred during the infusion with no significant AE in both dosage groups during 6 months follow-up, thus demonstrating that UCMSCs infusion was safe among healthy subjects. These outcomes are consistent with other UCMSCs treatment based studies where the group reported that the administration of UCMSCs with the best medical care was safe with reduced ejection fraction among patients with stable heart failure [22]. In addition, UCMSCs infusion posed no SAE among type 2 diabetes mellitus (T2DM) patients with mild improvement in hemoglobin A1c (HbA1c) and fasting blood sugar (FBS) [23].

Apart from assessing the safety of CLV-100 infusion, the outcome of the routine blood tests, lung function tests, and vital parameters in this Phase 1 study demonstrated MSCs' tolerability in allogeneic treatment as it did not trigger or increase hypersensitivity reactions in both LD and HD groups, which were maintained within the normal range throughout the follow-up period. It has been reported that MSCs administered in patients with moderate-to-severe atopic dermatitis (AD) could reduce allergic symptoms and inflammatory parameters via reduction of serum immunoglobulin E (IgE) levels and eosinophil count without the occurrence of serious AE [24]. We did not demonstrate the decrease in IgE and total white cell count. This is because our study involved healthy volunteers, and it also reaffirms that while MSCs are immunomodulatory, they are not immunosuppressive.

In terms of safety and tolerability, our lung function tests upon CLV-100 administration in both LD and HD groups were consistent with the previous study where MSCs infusion via endobronchial valve (EV) placement was well-tolerated and appear to decrease systemic inflammation in patients with compromised lung function due to severe chronic obstructive pulmonary disease (COPD) without any occurrence of acute administration-related toxicity, SAE, or death reported [25]. These results also proved where lung function test results, gas exchange variables, and blood work obtained after MSCs infusion have no significant alterations with those values obtained before MSCs infusion among patients with bronchiolitis obliterans syndrome [26].

We also found that high-dose CLV-100 infusion provided a significant increase in both hemoglobin level and MCV level that falls within the normal range. Another study indicated that MSCs maintained a better quality of hemoglobin as well as the oxygen-carrying capacity [27] as more hemoglobin helps in controlling the level of nitric oxide, thus expanding the blood vessels for more blood flow. Hemoglobin is also important for immunity where free hemoglobin serves as an alarm molecule that signals bleeding and tissue damage, which drives macrophage production towards a protective, antioxidative macrophage type, that halts lesion progression at later stages of disease [28]. In recent studies, patients with anaemia condition or with inflammatory diseases such as rheumatoid arthritis (RA) frequently have a significant reduction in hemoglobin and MCV level as compared to healthy or nonanaemic individuals [29, 30]; thus, these indirectly show the importance of MSCs and hemoglobin level towards immunity.

C-reactive protein (CRP) is a nonspecific proinflammatory marker that is commonly produced by the human body under stress condition as a systemic response towards acute infections, inflammatory conditions, and trauma. It plays a role in the recruitment of monocytes, granulocytes, and cytokines to the area of injury and infection to control the damage and initiate repair. As the injury is contained and healing begins, the hs-CRP starts to fall. Persistent hs-CRP, on the other hand, would have indicated or resulted in impaired healing and scarring and may be a risk factor for cardiovascular disease (CVD) [31]. Our results exhibited significant differences in hs-CRP level at different time points in both dosage groups where it was elevated initially followed by gradual declining trend within 6 months.

A similar finding was observed in another study where the hs-CRP and other proinflammatory cytokines including TNF-*α* and IL-6 raised at 7 days post-MSCs transplantation before receding within 1 month follow-up ([32]). Besides, they reported that the level of IL-10, an anti-inflammatory marker, also peaked at 7 days post-MSCs transplantation which then helped to reduce tissue inflammation after an injury caused by the previously mentioned proinflammatory cytokines [33]. Based on our results, the hs-CRP level was only peaked at 2 days post-MSCs infusion, which later followed by the raised level of IL-10, which peaked on the same day to ease down the inflammation rate even it was not statistically significant. This outcome was also supported by Jiao and colleagues where the levels of CRP and IL-10 were positively correlated among patients with traumatic fracture of lower limb [34].

In addition, the biomarkers assessment results showed a significant steady increase of cytokine IL-1RA level from baseline up until 6 months of posttreatment in the HD group. IL-1RA is a naturally occurring antagonist to the proinflammatory cytokine IL-1 especially IL-1*β* [35]. It has been reported that elevation in IL-1*β* activity triggered the increasing level of hs-CRP synthesized in the liver, making hs-CRP to become a surrogate biomarker for IL-1*β* [36–38]. Hence, due to its anti-inflammatory property, the increasing level of IL-1RA in this study could be interpreted as a result of the immunoregulatory properties of MSCs to counter-regulate hs-CRP level which also found to be increased in this study. The result is supported by the previous study where hs-CRP and IL-1RA levels were observed to be positively correlated with depressive symptoms in patients with type 2 diabetes (T2D) [39].

Our study clearly demonstrated a difference in immunomodulatory effect between the high dose and low dose. The HD group showed a significantly greater reduction of proinflammatory cytokine TNF-*α* at day 2 of posttreatment as well as an increased level of anti-inflammatory cytokines, which are IL-6, IL1-RA, and IL-10 within 6 months follow-up in relation to those in LD group. Hence, CLV-100 dosage of 130 million cells or two million MSCs per kilogram of body weight represents the optimal dose level in overcoming inflammatory conditions by displaying the best improvement in all parameters tested, absence of side-effects, and SAE. Our findings were consistent with another study which also concluded UCMSCs dosage of 100 million cells as the optimal dose level in treating frailty disease [40].

Moreover, the data collected in this study suggested that the expression level of IL-6 can be considered as an anti-inflammatory cytokine, because its expression's pattern in HD group is parallel with other anti-inflammatory cytokines, IL-10 and IL-1RA. Although IL-6 possesses proinflammatory properties and often correlates with disease severity, it is also paradoxically linked to anti-inflammatory molecules via complex auto-inhibitory feedback mechanisms where IL-6 plays a protective role in ischemic events by reducing the level of proinflammatory cytokine with the assistance of anti-inflammatory compounds IL-10 in healthy individuals [41].

Lastly, this is the first study that reported the significant changes of A/G ratio and globulin between visits with readings still fall within a normal range in subjects receiving a higher dosage of UCMSCs infusion. Total serum protein test is a common procedure to be included for a health check-up to measure the amount of protein in the body where albumins, globulin, and A/G ratio are the main components to be tested. In this study, a significant reduction of globulin was observed throughout the study. Globulin plays an important role in immunity, and it is known that increases in serum globulins are associated with several immune-mediated diseases (such as rheumatoid diseases, chronic liver disease, nephrotic syndrome, diabetes mellitus, and cancer) as well as related to chronic inflammation [42–44]. Moreover, decrease in globulin level in this study reflects the significant rising level of A/G ratio, a combination of two independent prognostic factors: albumin and globulin. Other researchers examined the relationship between the changes of A/G ratio value with the incidence of chronic diseases in healthy populations, and their findings indicate that healthy subjects with low A/G ratio (<1.1) were found to be at risk for not only liver cancer or hematologic malignancies but all the other common cancers [44]. Besides that, higher A/G ratio due to lower level of globulin may indicate better nutrition, lower inflammation, lower autoimmune disease, and infection and may have a positive effect to the overall survival of patients with solid tumours [42].

## 5. Conclusion

In conclusion, intravenous allogeneic infusion of CLV-100 was safe, well-tolerated, and free from any concerning AE toward all subjects in both LD and HD groups. Despite the small numbers of subjects, we had demonstrated an initial transient proinflammatory effect followed by a significant and prolonged anti-inflammatory effect. This immunomodulatory effect at high dose was accompanied by beneficial increases in hemoglobin and A/G ratio and with no adverse changes in vital parameters and tests of hypersensitivity. Therefore, high doses of allogenic MSCs could help exert beneficial effects of repair and healing.

## Figures and Tables

**Table 1:** Key inclusion and exclusion criteria for the enrolled subjects.

Key inclusion criteria
(i) Men and women aged 40 years and older (ii) Subjects with normoglycemia (iii) Subjects with normotension (iv) Subjects with normal fasting lipid profile (v) Subject must understand patient information sheet and signed informed consent form

Key exclusion criteria
(i) Subject who has enrolled in another investigational drug trial or innovative therapeutics product-related trial or has completed the aforesaid within 3 months (ii) Subject with history of current or past use (within 1 year) of alcohol, smoking, or drug abuse (iii) Pregnant or nursing women (iv) Subject with known documented drug allergies (v) Subject who is required of the following medicines on a regular basis: anti-histamine, steroid, antibiotic, anti-inflammatory, immunosuppressant, and pain killer medications (vi) Subject who is currently on any hormone replacement or hormone suppressive therapy for any indication (vii) Subject with any acute or chronic infections or communicable diseases including hepatitis B, hepatitis C, or HIV (viii) Subject with any active or past history of neoplasia and primary hematological disease (ix) Subject with any renal impairment indicated by serum creatinine ≥120 *μ*mol or creatinine clearance <60 mL/min (x) Subject with any cardiovascular disease including documented coronary disease of more than 50% stenosis, angina, myocardial infarction, heart failure, stroke, transient ischemic attack, and/or peripheral artery disease (xi) Subject with any diabetes mellitus (xii) Subject with any liver impairment indicated by serum aspartate transaminase and alanine transaminase greater than 1.5 times upper limit normal (xiii) Subject with any chronic pulmonary or airways disease (xiv) Subject with any current or past history of mental illness or cognitive impairment.

**Table 2:** Clinical characteristics in recruited subjects during baseline assessment.

Parameter	Normal range	Total (*n* = 11)	Low dose (*n* = 5)	High dose (*n* = 6)	*p* value^a^
Age	—	55 ± 13	52 ± 14	57 ± 13	0.36
Body weight	—	59.6 ± 9.1	55.2 ± 9.4	63.3 ± 7.6	0.15
Male	—	4	2	2	0.82
Female	—	7	3	4	0.82
Routine blood tests					
WBC (×10^9^/L)	4.0-11.0	6.7 ± 0.9	6.0 ± 0.5	7.3 ± 0.9	**0.03** ^∗^
Hemoglobin (g/dL)	11.5-16.5	13.4 ± 1.4	13.0 ± 1.6	13.8 ± 1.2	0.20
HCT (%)	35-47	41.2 ± 3.7	40.4 ± 3.8	41.9 ± 3.8	0.41
MCV (fl)	76-96	86.1 ± 4.0	86.4 ± 5.5	86.0 ± 2.8	0.52
Platelet (×10^9^/L)	150-400	292 ± 55	292 ± 76	292.7 ± 38	0.72
Creatinine (*μ*mol/L)	44-97	69.2 ± 21.3	65.8 ± 27.3	72.0 ± 17.2	0.36
ESR (mm/hr)	0-20	17.5 ± 17.5	14.4 ± 12.2	20.0 ± 21.9	0.86
AST (IU/L)	0-40	22.2 ± 6.5	19.6 ± 2.6	24.3 ± 8.1	0.27
ALT (IU/L)	0-53	20.7 ± 13.6	16.2 ± 4.4	24.5 ± 17.8	0.52
Albumin (g/L)	30-50	43.6 ± 3.5	44.5 ± 4.4	42.8 ± 2.8	0.58
Globulin (g/L)	20-50	30.9 ± 2.1	30.3 ± 2.6	31.3 ± 1.6	0.46
A/G ratio	1.2-2.5	1.4 ± 0.2	1.5 ± 0.2	1.4 ± 0.2	0.40
Total cholesterol (mmol/L)	<5.2	5.5 ± 0.9	5.4 ± 0.4	5.5 ± 1.3	0.71
HbA1c (%)	3.0-6.0	5.5 ± 0.4	5.7 ± 0.3	5.2 ± 0.4	0.07
FBS (mmol/L)	3.9-5.6	4.7 ± 0.4	4.7 ± 0.3	4.7 ± 0.5	0.93
Insulin (mU/L)	3.0-25.0	6.1 ± 5.6	7.6 ± 8.4	4.9 ± 1.6	1.00
IGF-1(ng/mL)	87-238	166.4 ± 57.5	161 ± 66.2	169.6 ± 59.6	0.88
DHEAS (*μ*mol/L)	1.0-11.7	3.0 ± 2.3	1.5 ± 1.5	3.8 ± 2.3	0.10
Estradiol (pg/mL)	50-100	66.5 ± 79.1	78.9 ± 107.2	54.0 ± 60.7	0.83
Progesterone (ng/mL)	0.57-6.11	2.3 ± 5.1	0.29 ± 0.1	4.4 ± 7.3	0.82
Testosterone (ng/mL)	2.41-8.27	3.8 ± 2.0	-^b^	3.8 ± 2.0	-^b^
hs-CRP (mg/L)	<4.7	1.1 ± 1.5	0.6 ± 0.5	1.6 ± 1.9	0.58
IgE (IU/mL)	<158.0	38.4 ± 42.5	23.3 ± 19.2	49.7 ± 54.5	0.48
Total PSA (ng/mL)	0.0-4.0	1.8 ± 0.7	1.2 ± 0.6	2.3 ± 0.1	0.12
CA125 (U/mL)	<35.0	9.7 ± 4.8	12.8 ± 6.4	7.4 ± 1.6	0.08
CA15.3 (U/mL)	<28.0	8.2 ± 4.5	8.3 ± 4.2	8.2 ± 5.4	0.72
CEA (ng/mL)	<5.0	0.9 ± 0.5	0.8 ± 0.3	1.0 ± 0.6	1.00
CA19.9 (U/mL)	<37.0	19.4 ± 9.1	22.1 ± 7.7	17.1 ± 10.3	0.36
AFP (ng/mL)	<15.0	3.1 ± 2.1	1.7 ± 0.4	4.2 ± 2.3	**0.04** ^∗^
Vital signs					
SBP (mmHg)	<129	121 ± 12	116 ± 8	125 ± 14	0.14
DBP (mmHg)	<80	75 ± 5	76 ± 6	75 ± 5	0.71
Heart rate (beats/min)	60-100	72 ± 9	73 ± 14	70 ± 5	0.27
Lung function tests					
FEV1 (L)	2.5-4.5	2.5 ± 0.5	2.6 ± 0.6	2.4 ± 0.4	0.36
FVC (L)	2.5-4.5	2.8 ± 0.6	2.9 ± 0.9	2.8 ± 0.3	0.86
FEV1/FVC (%)	>75	89 ± 7	92 ± 6	86 ± 7	0.20
Biomarkers					
IL-6 (pg/mL)	—	16 ± 5	18 ± 6	15 ± 3	0.36
IL-10 (pg/mL)	—	269 ± 64	289 ± 42	258 ± 74	0.30
PGE2 (pg/mL)	—	2947 ± 1417	3795 ± 2017	2523 ± 969	0.44
IL1-RA (pg/mL)	—	440 ± 117	447 ± 117	436 ± 128	0.80
TNF-*α* (pg/mL)	—	96 ± 29	118 ± 10	85 ± 30	0.12
TGF-*β* (ng/mL)	—	37 ± 5	35 ± 6	37 ± 4	0.80
VEGF (pg/mL)	—	528 ± 383	475 ± 281	556 ± 448	1.00
HGF (pg/mL)	—	855 ± 344	678 ± 254	944 ± 368	0.20

^a^The Mann–Whitney test. ^b^Data too low to be computed. ^∗^Significant value at *p* < 0.05. Abbreviation: WBC: white blood cells; MCV: mean corpuscular volume; HCT: hematocrit; A/G: albumin/globulin; ESR: erythrocyte sedimentation rate; AST: aspartate aminotransferase; ALT: alanine transaminase; HbA1c: hemoglobin A1c; FBS: fasting blood sugar; IGF-1: insulin growth factor-1; DHEAS: dehydropiandrosterone sulphate; SBP: systolic blood pressure; DBP: diastolic blood pressure; FEV1: forced expiratory volume in one second; FVC: forced vital capacity; hs-CRP: high-sensitivity C-reactive protein; IgE: immunoglobulin E; IL-6: interleukin 6; IL-10: interleukin 10; PGE2: prostaglandin E2; IL1-RA: interleukin 1 receptor antagonist; TNF-*α*: tumor necrosis factor-alpha.

**Table 3:** Baseline and follow-up clinical assessments among subjects in the LD group.

Parameters	Baseline day 0	Follow-up period				*p* value^a^
		Day 2	Day 30	Day 90	Day 180	
Routine blood tests						
WBC (×10^9^/L)	6.0 ± 0.5	5.4 ± 0.5	5.6 ± 1.3	5.4 ± 1.0	5.8 ± 1.1	0.51
Hemoglobin (g/dL)	13.0 ± 1.6	12.9 ± 1.5	13.0 ± 1.5	13.0 ± 1.6	13.2 ± 1.1	0.74
Creatinine (umol/L)	65.8 ± 27.3	62.6 ± 15.9	66.4 ± 24.0	64.6 ± 14.9	62.6 ± 13.9	0.97
HCT (%)	40.4 ± 3.8	40.0 ± 4.1	40.2 ± 4.1	40.6 ± 4.3	41.4 ± 2.9	0.59
MCV (fl)	86.4 ± 5.5	86.0 ± 4.3	86.2 ± 4.5	85.6 ± 5.4	85.8 ± 4.9	0.72
Platelet (×10^9^/L)	292 ± 77	282 ± 74	282 ± 106	275 ± 3	298 ± 99	0.96
ESR (mm/hr)	14.4 ± 12.2	16.6 ± 11.4	21.6 ± 20.4	15.6 ± 9.5	12.6 ± 6.6	0.69
AST (IU/L)	19.6 ± 2.6	21.6 ± 3.3	20.2 ± 2.2	22.6 ± 4.6	21.4 ± 3.2	0.34
ALT (IU/L)	16.2 ± 4.4	16.2 ± 5.9	16 ± 3.9	17.2 ± 4.7	17.4 ± 1.3	0.92
Albumin (g/L)	44.5 ± 4.4	42.2 ± 3.2	42.2 ± 3.8	43.2 ± 3.5	41.6 ± 2.1	0.07
Globulin (g/L)	30.3 ± 2.6	31.2 ± 2.3	31.2 ± 3.5	31.2 ± 4.2	30.8 ± 2.9	0.96
A/G ratio	1.5 ± 0.2	1.3 ± 0.2	1.4 ± 0.2	1.4 ± 0.2	1.4 ± 0.2	0.06
Total cholesterol (mmol/L)	5.4 ± 0.4	5.2 ± 0.3	5.1 ± 0.5	5.4 ± 0.6	5.3 ± 0.6	0.59
HbA1c (%)	5.7 ± 0.3	—	5.7 ± 0.4	5.7 ± 0.3	5.6 ± 0.3	0.78
FBS (mmol/L)	4.7 ± 0.3	—	4.8 ± 0.3	4.7 ± 0.4	4.6 ± 0.3	0.64
Insulin (mU/L)	7.6 ± 8.4	—	4.4 ± 2.6	2.9 ± 2.0	3.8 ± 0.3	0.68
IGF-1 (ng/mL)	161 ± 66.2	—	123.4 ± 27.4	127.9 ± 44.0	133.2 ± 58.1	0.62
DHEAS (*μ*mol/L)	1.5 ± 1.5	—	1.1 ± 1.1	1.4 ± 1.1	1.2 ± 0.7	0.46
Estradiol (pg/mL)	78.9 ± 107.2	—	85.9 ± 123.3	51.7 ± 40.2	62.4 ± 71.9	0.90
Progesterone (ng/mL)	0.29 ± 0.1	—	7.3 ± 12.3	0.2 ± 0.0	5.7 ± 9.7	0.52
Testosterone (ng/mL)	-^b^	—	-^b^	-^b^	-^b^	-^b^
hs-CRP (mg/L)	0.6 ± 0.5	3.5 ± 3.2	2.1 ± 3.3	1.1 ± 1.1	1.5 ± 1.8	**0.03** ^∗^
IgE (IU/mL)	23.3 ± 19.2	23.3 ± 18.4	24.6 ± 19.6	20.9 ± 16.6	22.1 ± 12.0	0.14
Total PSA (ng/mL)	1.2 ± 0.6	—	1.3 ± 1.0	1.2 ± 0.8	1.2 ± 0.9	0.90
CA125 (U/mL)	12.8 ± 6.4	—	11.6 ± 4.5	12.4 ± 5.5	11.3 ± 3.4	0.90
CA15.3 (U/mL)	8.3 ± 4.2	—	9.5 ± 5.4	8.6 ± 4.0	8.6 ± 3.0	0.82
CEA (ng/mL)	0.8 ± 0.3	—	1.1 ± 0.5	0.8 ± 0.3	0.9 ± 0.3	0.23
CA19.9 (U/mL)	22.1 ± 7.7	—	19.4 ± 11.3	19.5 ± 10.2	18.3 ± 9.4	0.52
AFP (ng/mL)	1.7 ± 0.4	—	1.3 ± 0.1	2.2 ± 0.9	1.5 ± 0.3	0.14
Vital signs						
SBP (mmHg)	116 ± 8	119 ± 19	121 ± 18	120 ± 11	117 ± 16	0.89
DBP (mmHg)	76 ± 6	73 ± 8	75 ± 8	78 ± 6	75 ± 6	0.21
Heart rate (beats/min)	73 ± 14	66 ± 10	72 ± 12	71 ± 11	68 ± 13	0.40
Lung function tests						
FEV1 (L)	2.6 ± 0.6	—	—	2.5 ± 0.5	2.3 ± 0.5	0.13
FVC (L)	2.9 ± 0.9	—	—	2.9 ± 0.5	2.7 ± 0.7	0.82
FEV1/FVC (%)	92 ± 6	—	—	87 ± 6	89 ± 9	0.25
Biomarkers						
IL-6 (pg/mL)	18 ± 6	19 ± 14	10 ± 4	—	12 ± 4	0.56
IL-10 (pg/mL)	289 ± 42	271 ± 37	231 ± 13	—	251 ± 28	0.21
PGE2 (pg/mL)	3795 ± 2017	3198 ± 1076	2431 ± 923	—	3206 ± 1533	0.76
IL1-RA (pg/mL)	447 ± 117	415 ± 126	434 ± 225	—	306 ± 36	0.22
TNF-*α* (pg/mL)	118 ± 10	115 ± 15	93 ± 16	—	78 ± 3	0.06
TGF-*β* (ng/mL)	35 ± 6	33 ± 11	28 ± 6	—	35 ± 6	0.54
VEGF (pg/mL)	475 ± 281	648 ± 273	723 ± 258	—	773 ± 258	0.27
HGF (pg/mL)	678 ± 254	859 ± 169	1268 ± 307	—	1037 ± 170	0.13

^a^The Friedman test. ^b^Data too low to be computed. ^∗^Significant value at *p* < 0.05.

**Table 4:** Baseline and follow-up clinical assessments among subjects in the HD group.

Parameters	Baseline day 0	Follow-up period				*p* value^a^
		Day 2	Day 30	Day 90	Day 180	
Routine blood tests						
WBC (×10^9^/L)	7.3 ± 0.9	6.4 ± 0.7	6.6 ± 0.9	6.6 ± 1.3	6.8 ± 0.7	0.69
Hemoglobin (g/dL)	13.8 ± 1.2	13.6 ± 1.4	13.5 ± 1.1	14.1 ± 1.1	14.4 ± 1.3	**0.04** ^∗^
HCT (%)	41.9 ± 3.8	42.0 ± 4.2	42.0 ± 2.9	43.0 ± 3.3	43.7 ± 3.9	0.12
MCV (fl)	86.0 ± 2.8	87.5 ± 3.0	87.5 ± 3.7	88.3 ± 3.3	88.2 ± 3.9	**0.02** ^∗^
Platelet (×10^9^/L)	293 ± 39	286 ± 36	297 ± 30	299 ± 26	298 ± 34	0.20
Creatinine (*μ*mol/L)	72.0 ± 17.2	68.8 ± 21.6	70.5 ± 20.2	67.8 ± 13.9	74.7 ± 14.1	0.50
ESR (mm/hr)	20.0 ± 21.9	21.0 ± 16.4	19.5 ± 17.4	17.2 ± 16.0	18.8 ± 19.2	0.13
AST (IU/L)	24.3 ± 8.1	23.3 ± 6.3	23.0 ± 6.4	25.3 ± 4.3	24.2 ± 5.3	0.81
ALT (IU/L)	24.5 ± 17.8	23.3 ± 12.8	21.0 ± 10.3	24.3 ± 13.6	23.2 ± 10.6	0.77
Albumin (g/L)	42.8 ± 2.8	43.2 ± 3.9	43.5 ± 2.9	43.5 ± 2.4	44.2 ± 2.9	0.23
Globulin (g/L)	31.3 ± 1.6	29.5 ± 1.6	28.5 ± 1.2	28.3 ± 1.6	27.8 ± 1.6	**0.01** ^∗^
A/G ratio	1.4 ± 0.2	1.5 ± 0.1	1.5 ± 0.1	1.6 ± 0.1	1.6 ± 0.1	**0.01** ^∗^
Total cholesterol (mmol/L)	5.5 ± 1.3	5.3 ± 1.0	5.3 ± 0.9	5.4 ± 0.7	6.1 ± 1.3	0.09
HbA1c (%)	5.2 ± 0.4	—	5.5 ± 0.3	5.5 ± 0.4	5.6 ± 0.4	0.08
FBS (mmol/L)	4.7 ± 0.5	—	4.4 ± 0.5	4.4 ± 0.3	4.6 ± 0.3	0.20
Insulin (ng/mL)	4.9 ± 1.6	—	5.2 ± 1.8	5.7 ± 3.6	4.3 ± 2.6	0.14
IGF-1(ng/mL)	169.6 ± 59.6	—	156.4 ± 69.1	155.6 ± 83.3	148.8 ± 44.9	0.78
DHEAS (*μ*mol/L)	3.8 ± 2.3	—	3.2 ± 2.5	3.7 ± 2.6	3.4 ± 2.3	0.12
Estradiol (pg/mL)	54.0 ± 60.7	—	38.6 ± 35.4	66.7 ± 55.0	111.0 ± 87.1	0.15
Progesterone (ng/mL)	4.4 ± 7.3	—	0.9 ± 1.3	0.3 ± 0.1	7.1 ± 12.0	0.86
Testosterone (ng/mL)	3.8 ± 2.0	—	3.8 ± 1.1	5.5 ± 0.5	4.1 ± 1.9	0.62
hs-CRP (mg/L)	1.6 ± 1.9	5.3 ± 4.5	1.6 ± 1.6	2.7 ± 2.1	1.8 ± 2.3	**0.04** ^∗^
IgE (IU/mL)	49.7 ± 54.5	46.8 ± 48.4	48.6 ± 52.5	46.1 ± 49.2	50.2 ± 53.3	0.79
Total PSA (ng/mL)	2.3 ± 0.1	—	1.4 ± 0.8	1.4 ± 0.9	1.5 ± 1.0	0.24
CA125 (U/mL)	7.4 ± 1.6	—	7.6 ± 2.8	7.3 ± 1.4	6.5 ± 1.9	0.96
CA15.3 (U/mL)	8.2 ± 5.4	—	8.1 ± 6.1	8.9 ± 7.2	9.6 ± 6.1	0.31
CEA (ng/mL)	1.0 ± 0.6	—	0.9 ± 0.4	0.9 ± 0.5	1.1 ± 0.9	0.21
CA19.9 (U/mL)	17.1 ± 10.3	—	17.0 ± 9.0	15.0 ± 9.1	13.6 ± 11.0	0.06
AFP (ng/mL)	4.2 ± 2.3	—	4.2 ± 2.7	3.8 ± 2.2	4.3 ± 3.1	0.88
Vital signs						
SBP (mmHg)	125 ± 14	120 ± 12	118 ± 17	131 ± 14	125 ± 11	0.16
DBP (mmHg)	75 ± 5	77 ± 5	74 ± 9	76 ± 9	77 ± 5	0.21
Heart rate (beats/min)	70 ± 5	70 ± 3	67 ± 5	71 ± 10	66 ± 2	0.39
Lung function tests						
FEV1 (L)	2.4 ± 0.4	2.3 ± 0.1	—	2.3 ± 0.2	2.3 ± 0.2	0.79
FVC (L)	2.8 ± 0.3	2.7 ± 0.3	**—**	2.8 ± 0.3	2.8 ± 0.3	**0.03** ^∗^
FEV1/FVC (%)	86 ± 7	84 ± 7	—	82 ± 4	83 ± 6	0.95
Biomarkers						
IL-6 (pg/mL)	14 ± 4	13 ± 4	22 ± 7	—	23 ± 5	0.06
IL-10 (pg/mL)	258 ± 74	215 ± 39	307 ± 49	—	321 ± 27	0.13
PGE2 (pg/mL)	2523 ± 969	4987 ± 4059	3208 ± 853	—	3378 ± 691	0.19
IL1-RA (pg/mL)	436 ± 128	610 ± 176	615 ± 148	**—**	705 ± 160	**0.03** ^∗^
TNF-*α* (pg/mL)	84 ± 30	74 ± 23	71 ± 15	—	67 ± 19	0.67
TGF-*β* (pg/mL)	37 ± 4	38 ± 5	34 ± 9	—	37 ± 8	0.42
VEGF (pg/mL)	556 ± 448	510 ± 134	599 ± 335	—	515 ± 297	0.58
HGF (pg/mL)	944 ± 368	1152 ± 436	1174 ± 539	—	1124 ± 628	0.58

^a^The Friedman test. ^∗^Significant value at *p* < 0.05.

## Data Availability

The datasets used and/or analyzed during the study are available from the first author upon request.
